# Effect of the explicit flexibility of the InhA enzyme from *Mycobacterium tuberculosis* in molecular docking simulations

**DOI:** 10.1186/1471-2164-12-S4-S7

**Published:** 2011-12-22

**Authors:** Elisangela ML Cohen, Karina S Machado, Marcelo Cohen, Osmar Norberto de Souza

**Affiliations:** 1 Laboratório de Bioinformática, Modelagem e Simulação de Biossistemas - LABIO, Programa de Pós-Graduação em Ciência da Computação - PPGCC - Faculdade de Informática - PUCRS - Av. Ipiranga, 6681 Prédio 32 - Sala 608. CEP: 90619-900, Porto Alegre, RS, Brasil; 2 Programa de Pós-Graduação em Biologia Celular e Molecular - PPGBCM - Faculdade de Biociências - PUCRS - Av. Ipiranga, 6681 Prédio 12. CEP: 90619-900, Porto Alegre, RS, Brasil

## Abstract

**Background:**

Protein/receptor explicit flexibility has recently become an important feature of molecular docking simulations. Taking the flexibility into account brings the docking simulation closer to the receptors’ real behaviour in its natural environment. Several approaches have been developed to address this problem. Among them, modelling the full flexibility as an ensemble of snapshots derived from a molecular dynamics simulation (MD) of the receptor has proved very promising. Despite its potential, however, only a few studies have employed this method to probe its effect in molecular docking simulations. We hereby use ensembles of snapshots obtained from three different MD simulations of the InhA enzyme from *M. tuberculosis* (Mtb), the wild-type (InhA_wt), InhA_I16T, and InhA_I21V mutants to model their explicit flexibility, and to systematically explore their effect in docking simulations with three different InhA inhibitors, namely, ethionamide (ETH), triclosan (TCL), and pentacyano(isoniazid)ferrate(II) (PIF).

**Results:**

The use of fully-flexible receptor (FFR) models of InhA_wt, InhA_I16T, and InhA_I21V mutants in docking simulation with the inhibitors ETH, TCL, and PIF revealed significant differences in the way they interact as compared to the rigid, InhA crystal structure (PDB ID: 1ENY). In the latter, only up to five receptor residues interact with the three different ligands. Conversely, in the FFR models this number grows up to an astonishing 80 different residues. The comparison between the rigid crystal structure and the FFR models showed that the inclusion of explicit flexibility, despite the limitations of the FFR models employed in this study, accounts in a substantial manner to the induced fit expected when a protein/receptor and ligand approach each other to interact in the most favourable manner.

**Conclusions:**

Protein/receptor explicit flexibility, or FFR models, represented as an ensemble of MD simulation snapshots, can lead to a more realistic representation of the induced fit effect expected in the encounter and proper docking of receptors to ligands. The FFR models of InhA explicitly characterizes the overall movements of the amino acid residues in helices, strands, loops, and turns, allowing the ligand to properly accommodate itself in the receptor’s binding site. Utilization of the intrinsic flexibility of Mtb’s InhA enzyme and its mutants in virtual screening via molecular docking simulation may provide a novel platform to guide the rational or dynamical-structure-based drug design of novel inhibitors for Mtb’s InhA. We have produced a short video sequence of each ligand (ETH, TCL and PIF) docked to the FFR models of InhA_wt. These videos are available at http://www.inf.pucrs.br/~osmarns/LABIO/Videos_Cohen_et_al_19_07_2011.htm.

## Background

Molecular docking simulation constitutes one of the main stages of rational or structure-based drug design [1]. It provides a prediction for a molecule binding to a protein in order to form a stable complex [2]. Knowledge of proper orientation can be used to predict the strength of association or binding affinity between two molecules. Initially, molecular docking was compared to the classic "key-lock” theory of enzyme-substrate specificity postulated by Emil Fischer in 1894 (Reviewed by Koshland Jr., [3,4]). In this model, the three-dimensional (3-D) structure of both ligand and protein complement each other in the same way a key fits the corresponding lock [5]. However, since both protein and ligand are flexible molecules, the concept is no longer adequate as during the process of molecular docking both ligand and protein adjust their conformation in order to achieve the best protein-ligand fit. This type of conformational adjustment between the two molecules, or the induced fit theory, was first presented by Daniel E. Koshland Jr. in 1958 [3,4].

In order to make molecular docking simulations more realistic, an important issue is to treat both receptor and ligand as flexible structures instead of rigid bodies. In many methods the ligand, usually a small molecule with up to dozens of atoms, is treated as flexible, but the flexibility of the protein/receptor (for simplicity, herein protein and receptor are synonymous), depending on their complexity and size, which can reach dozens of thousands of atoms, is still treated in a more restricted manner. According to Cozzini et al. “the challenge for drug discovery, as well as docking or virtual screening, is to model the plasticity of the receptor so that both structures can conformationally adapt to each other” [6]. Therefore, it is well known in the literature that the recognition of the ligand by the receptor is a dynamic event, where both structures change their conformations to minimize the free energy of binding (FEB) for their association [7]. Nevertheless, most methods of docking employ a single, rigid structure of the receptor. This happens for practical reasons. If we try to consider the explicit flexibility of receptor and ligand, the conformational space to be considered quickly becomes impractical [8,9], as the process would require an enormous computational effort. In addition, Totrov and Abagyan [10] state that the best docking algorithms today erroneously predict the position of ligand binding in 50 to 70% of the cases, when only one receptor conformation is considered.

In biological systems, proteins express their functions in aqueous or semi-fluid environments. When in solution, proteins exist in a number of energetically different conformations, so that their structure is best described when all the different states are represented [6]. A set of structures of a particular protein can be determined experimentally by X-ray crystallography or Nuclear Magnetic Resonance, through computational methods which includes Monte Carlo and molecular dynamics (MD) simulations [11]. Therefore if we consider the explicit flexibility using multiple receptor conformations, there are also a number of approaches. An example is the relaxed complex scheme (RCS) [12]. The idea is to perform MD simulation of the unliganded receptor before docking to address its flexibility. The RCS method acknowledges that a ligand probably will bind to conformations of the receptor that occur rarely in its dynamic state. This strong binding often indicates multivalent attachment of the ligand to the receptor. The second phase of the RCS method involves the rapid docking of small libraries of ligands to a large ensemble of MD-derived receptor conformations. Further information and comprehensive reviews of different methods for flexible-receptor flexible-ligand docking can be found in [13-17].

There have been very few studies on the effect of MD-derived receptor flexibility in flexible-ligand docking simulations [14,16-18]. In order to improve the body of evidence about the role that receptor flexibility plays in molecular docking simulations we use ensembles of snapshots obtained from three different MD simulations of the InhA enzyme from Mtb; InhA_wt, and the mutants InhA_I16T, and InhA_I21V. Each ensemble of snapshots is denominated a fully-flexible receptor (FFR) model. We have used them to systematically explore their docking to three different InhA inhibitors, namely, ethionamide (ETH), triclosan (TCL), and pentacyano(isoniazid)ferrate(II) (PIF).

## Methods

In order to carry out docking simulations, we need a receptor model, and at least one ligand, as well as docking software. Below we describe the different steps involved in processing a docking simulation, including the definition of the rigid and flexible receptor models, the preparation of the ligands, particularly the reference ligands’ position that will be used in our supervised docking simulations.

### The single-rigid receptor model

**Figure 1:**
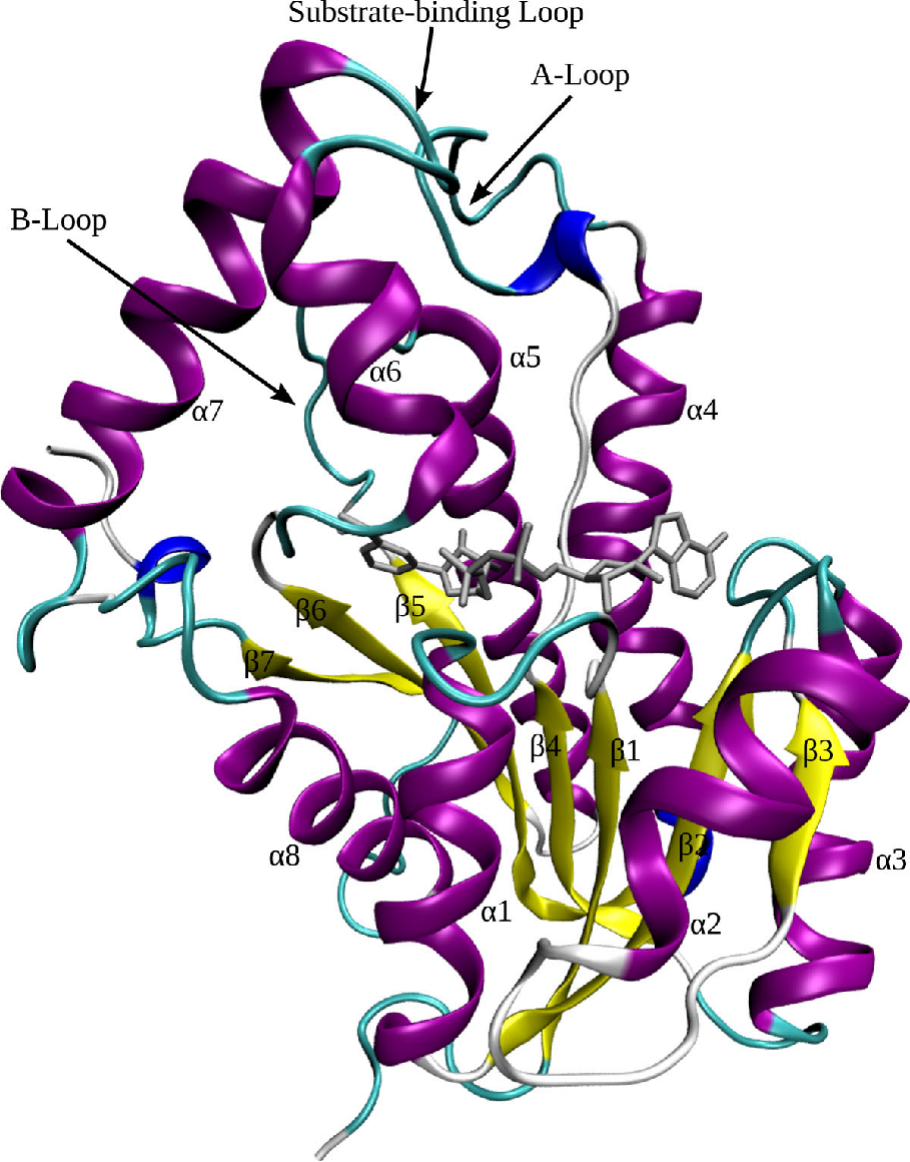
**The InhA tertiary structure.** Ribbons representation of Mtb’s InhA (PDB ID:1ENY) rigid receptor model (colored by secondary structure) in complex with the coenzyme NADH (in metallic grey). In yellow are the 7-strands parallel β-sheet and in magenta the eight α-helices, connected by loops (in cyan) and turns (in white). The protein’s N-terminus is composed by helices α1 and α2, and by the β1 to β3 strands, while the C-terminus is formed by helices α7 and α8. Figure produced with VMD [29].

The InhA enzyme or 2-*trans*-Enoyl-ACP (CoA) reductase (EC number 1.3.1.9) from *M. tuberculosis* was chosen as receptor model for this work because of its importance as a drug target against tuberculosis [19]. It belongs to the SDR (short chain dehydrogenase / reductase) family of proteins, which uses NADH (β-nicotinamide adenine dinucleotide, reduced form) as coenzyme. The main feature of this family is the topology of the polypeptide backbone, where each subunit of the protein is composed of a single domain with a classical Rossmann-fold topology [20]. It is characterized by a 7-strands parallel β-sheet and eight α-helices, connected by loops and turns, forming the NADH binding site (Figure 1). The enzyme has a “chair-like” appearance where the “legs” and “backrest” are topologically similar to other dehydrogenases. The substrate binding cavity is a “pocket” located in the backrest. It is formed by the substrate-binding loop (helices α6 and α7 in Figure 1), strands β4, β5, and helix α5 [20]. The NADH coenzyme is positioned in an extended conformation in the pocket along the top of the parallel β-sheet. The adenine ring is nearly parallel to the “seat” of the structure while the nicotinamide portion faces backwards, pointing to the base of the substrate binding cavity [20]. The A- and B-loops, as well as the substrate-binding loop are structural motifs key to this enzyme function. The rigid model of InhA is taken from its crystal structure (PDB ID: 1ENY) and will be called 1ENY throughout the text.

### The Fully-Flexible Receptor (FFR) models

In this study we have considered three FFR models of Mtb’s InhA enzyme: (1) the InhA_wt, (2) the InhA_I16T mutant, and (3) the InhA_I21V mutant. All three FFR models were built from MD simulations of the InhA_wt-NADH (3.1 ns), InhA_I16T-NADH (5.5 ns) and InhA_I21V-NADH (6.5 ns) complexes generated with the SANDER module of AMBER 6 [21]. The mutants were constructed from the 1.9 ns instantaneous snapshot of the InhA_wt MD simulation [21]. These mutations have been clinically associated with isoniazid drug resistance [22]. Schroeder et al. [21] showed that the resistance was due to a reduced affinity of the NADH for the InhA enzyme. Because ETH action is similar to that of isoniazid, we expect a difference in its binding to both the InhA_wt and InhA mutants. The MD trajectories of the mutants and wild type simulations had different lengths. However, to have coherent flexible models, we considered 3.1 ns of each MD simulation. For instance, each mutant FFR model was generated from a trajectory in the interval ranging from 1.9 ns to 5.0 ns. We used intervals of 1.0 ps. Accordingly, each FFR model is composed of 3,100 snapshots.

#### Receptor preparation

Each FFR model of the InhA enzyme, which is an ensemble of snapshots from its MD simulation, was converted into the conventional PDB file using the *Ptraj* module of AMBER 9 [23]. *Ptraj* also computed an average structure for each FFR model considering the production phase of each trajectory, the last 1,000 snapshots. The FFR models were then fitted to their average structures, as well as to the rigid model 1ENY. In this manner, all snapshots making up each FFR model, the average structure and the rigid model of InhA will be all in the same frame of reference. Finally, we added the appropriate partial atomic charges, and solvation parameters using the *addsol* module of AutoDock 3.0.5 [24], to each receptor model. All the steps described above were accomplished with the scientific workflow described in [25].

### The ligands

**Figure 2:**
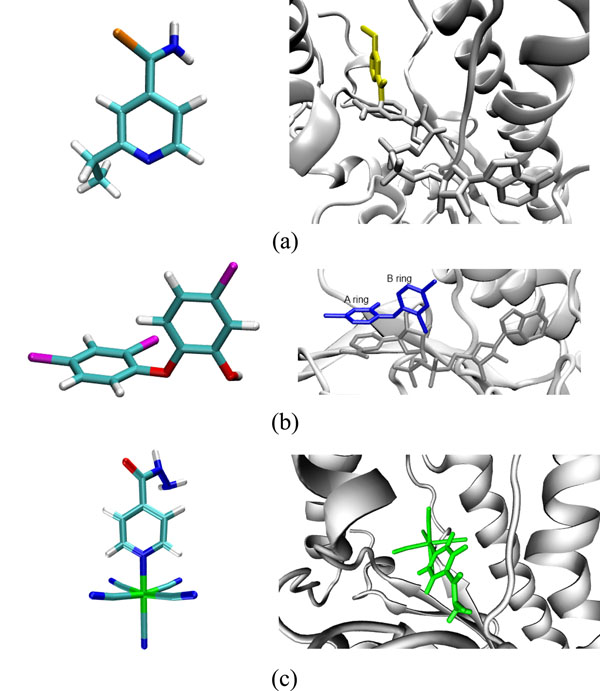
**The ligands.** (A) On the left, stick model representation of the ETH ligand. The hydrogen, nitrogen, carbon, and sulphur atoms are colored white, dark blue, cyan, and light brown, respectively. In the right, stick model representation of the adduct ETH-NAD (PDB ID: 2H9I). Activated ETH is colored in yellow and NAD in metallic grey. The adduct binds to the InhA active site with ETH blocking part of the substrate binding cavity. (B) Stick model representation of the TCL ligand. The color scheme and receptor representation are the same as in (A), except for oxygen and chlorine which are colored red and magenta, respectively. Stick model representation of the π stacking interaction between TCL’s A ring (dark blue) and the nicotinamide ring of NADH (from PDB ID: 1P45). (C) Stick model representation of the PIF ligand. Here the iron atom is colored green. Stick model representation of one possible interaction between PIF (green) and the InhA enzyme. InhA main-chain is represented by ribbons.

Ethionamide (ETH) (ZINC code: 3872520, accessed on 08/10/2010) is a relatively small molecule, composed of 21 atoms (Figure 2a). This is a powerful second line tuberculostatic, an isoniazid (INH) structural analogue, and is widely used in the treatment of tuberculosis because its primary target is the InhA protein. Like INH, ETH is also a pro-drug that requires prior activation [26]. Its mode of action is similar to that of INH. ETH binds covalently to carbon 4 of the nicotinamide portion of NADH to form the adduct ETH-NAD (Figure 2a).

Triclosan (TCL), (ZINC code: 2216, accessed on 08/10/2010) is composed of 24 atoms grouped into two aromatic rings (Figure 2b). It is an antibacterial and antifungal agent commonly found in various preparations ranging from toothpaste, cosmetics in general, antiseptic soaps and even plastic. In 1998, McMurry, Oethinger and Levy [27] suggested for the first time that TCL blocked the biosynthesis of fatty acids by inhibiting the enoyl reductase (ENR) or InhA. The TCL phenolic ring (A ring in Figure 2b) forms π-stacking interactions with the nicotinamide ring of NADH. Such interactions are formed due to stacking of aromatic rings of different molecules through van der Waals forces [28].

Pentacyano(isoniazid)ferrate(II) (PIF) is the result of a rational drug design effort by Santos, Basso and co-workers [29] in an attempt to find new inhibitors for *M. tuberculosis*’ InhA enzyme that do not require activation. This is an INH molecule with a metallic centre, the pentacyanoferrate group, bound to it (Figure 2c). The PIF molecule is composed of 28 atoms. Since the crystal structure of the InhA-PIF complex is yet not available, we performed molecular docking simulations to predict the binding mode of PIF to InhA [29].

#### Definition of the reference ligands

In many docking simulations described in the literature, the approach chosen is blind docking, where the ligand is placed at an initial arbitrary position within the active site of the target receptor, and from there, the docking software seeks to find the best ligand orientation that should correspond to the most negative FEB. The docking results are presented in histograms (Table 1). The problem is that the most negative estimated FEB does not necessarily correspond to the actual binding mode (here we call Best Pose) of the ligand found in experimental results [26].

**Table 1:** A typical example of a RMSD (in Å) clustering analysis of a 10 “runs” docking simulation with AutoDock 3.0.5

Rank	Sub-Rank	Run	Docked energy	Cluster RMSD	Reference RMSD
1	1	1	-8.99	0.00	4.10
1	2	10	-8.92	0.30	4.12
1	3	4	-8.90	0.37	4.08
1	4	9	-8.90	0.37	4.09
1	5	5	-8.87	0.22	4.15
1	6	8	-8.44	1.00	3.92
2	1	7	**-8.27**	0.00	5.84
2	2	3	-7.92	0.46	5.71
3	1	6	-7.84	0.00	4.19
4	1	2	-7.80	0.00	5.70

Autodock clusters by first sorting all the docked conformations from lowest (Docked energy, fourth column) to highest energy (in kcal/mol). The best overall docked conformation is used as the ‘seed’ for the first cluster (Rank 1, first column). Then the coordinates of the second best conformation are compared with those of the best to calculate the root-mean square deviation (Cluster RMSD, fifth column) between the two conformations. If the calculated RMSD value is smaller than or equal the specified cut-off, which is 1.0 by default, that conformation is added to the ‘bin’ containing the best conformation. If not, the second becomes the reference for a second ‘bin’ (Rank 2, seventh row). In this example we have four “bins” or clusters. As can be seen from the table, the 10 “runs” generated four clusters. Cluster 1 contains six out of 10 “runs” and so forth. The last column shows the RMSD of the corresponding conformation with respect to a reference ligand position. The bold value is the best estimated docked energy corresponding to the best docked pose expected from experiments [25].

For this reason, before starting the docking simulations with the three different receptor models, we performed a blind docking simulation with the 1ENY structure and ETH in order to compare the best docking results with the experimental one from the crystal structure [26]. In fact, run 7 (Table 1, in bold) is the one that gives the best ligand pose of ETH in InhA. Note that its FEB value of -8.27 kcal/mol is greater than the best one (-8.99 kcal/mol) ranked automatically by the docking program.

From this test experiment, we decided not to perform blind docking, but supervised dockings instead. For each ligand, we first tried to find its conformation and orientation corresponding to the Best Pose**.** The coordinates for this ligand pose was then saved and used as a reference coordinate to calculate the reference root-mean-square deviation (RMSD) (Table 1) of the ligand in the docking simulations. The same process was repeated for TCL, and PIF. Thus, in the docking experiments in this study both, the FEB estimates and RMSD values will be important to describe the effect of the FFR models in docking simulations.

#### Ligand preparation

Using the VMD software [31], the ligands in the Tripos Mol2 file format were randomly positioned at the receptor binding site in the rigid model, as well as in the three InhA FFR models. Since all models are in the same frame of reference, the initial position of all three ligands is same in all docking simulations for all models. The ligand flexibility was determined by the *deftors* module of AutoDock 3.0.5. ETH and TCL, and PIF have two and three rotatable bonds, respectively.

### Molecular docking simulations

The docking simulations were performed with AutoDock 3.0.5 [32] which has been extensively tested and proved to be successful in a variety of docking experiments [14]. It can use various techniques to explore the different conformations a ligand can assume, combining the advantages of a complete search space and the assessment of the best FEB [32].

#### Docking simulation parameters

With the rigid 1ENY model and the three FFR models of InhA in the PDBQS format, the active site was defined within a grid of 100 x 60 x 60 points, spaced at 0.375 Å. This set up generated a grid box with approximately 37 x 22 x 22 Å^3^, centred in the initial position of the ligand. This grid is large enough to include the NADH, as well as the substrate-binding cavity of InhA. Each docking simulation was composed of 25 independent runs, for which a maximum number of 27,000 generations were produced, employing the Lamarckian Genetic Algorithm (LGA) on the initial population of 50 individuals, a maximum number of 500,000 energy evaluations, with an elitism value of 1, a mutation rate of 0.02, and a cross-over rate of 0.8. For the local search the pseudo-Solis and Wets method was applied using default parameters. Each run provides one predicted binding mode. At the end of the docking experiment binding modes with RMSD of 1.0 Å within each other were placed in the same cluster.

#### Control docking

We performed docking simulations as a control for each of the ligands using the 1ENY rigid model. All results found for the docked FFR models of InhA will be compared to these controls.

#### Receptor-ligand interaction analyses

After performing the docking simulations, we employed LIGPLOT 4.4.2 [33] to analyze the hydrogen bonds and hydrophobic contacts between the ligands and the rigid and FFR models of InhA_wt and the mutants InhA_I16T and InhA_ I21V. LIGPLOT defines a FFR residue to be in a hydrophobic contact (NNB) with a ligand if there is at least one heavy atom of the residue within 3.9 Å of some atom from the ligand. For hydrogen bonds (HHB) the criteria is more restricted. The donor and acceptor atoms of FFRs’ residues and ligands have to be at a maximum distance of 3.5 Å [33].

### Automating the molecular docking simulations

We carried out the molecular docking experiments in a Core 2 Quad 2.4 GHz machine, with 8 GB of RAM and 500 GB HD. However, as there is currently no reliable, automated way to perform docking simulations in FFR models as the ones used in this work, our solution was to create in-house processing scripts using the programming languages *Bash*, *Awk* and *Python*.

### Automating the docking analyses

In order to carry out the docking analyses with LIGPLOT [33], we also created processing scripts, which are detailed below.

1. For each docked snapshot in a given FFR-ligand complex we extracted and stored the best runs according to the lowest FEB (blind docking) and the lowest RMSD in separate tables. We note that this RMSD measure is calculated with respect to the reference ligand pose (Best Pose in the supervised docking). This produced nine tables (ETH, TCL, and PIF against each of the InhA_wt, InhA_ I16T and InhA_I21V FFR models).

2. We then ran LIGPLOT for each FFR-ligand complex, processing the output files in order to extract and store the amino acid contacts of each one into a secondary table. This step produced 36 tables (the previous nine combinations split into HHB and NNB intermolecular contacts).

3. Finally, we counted the amino acid contacts, producing extra 36 tables for the FFR-ligand complexes describe above. The goal here was to identify which residues of the FFR models had interacted most with the ligands.

## Results

Flexible ETH, TCL, and PIF ligands were docked to three different FFR models of Mtb’s InhA: InhA_wt, InhA_I16T and InhA_I21V. Each complex gave us a set of docking results composed of the average and standard deviations (SD) of the best FEB (in kcal/mol) and its corresponding RMSD (in Å) with respect to the reference pose for the ligand (Table 2, columns A and B). We also obtained similar statistics for the set of FEB values matching the lowest RMSD with respect to the reference pose for the ligands (Table 2, columns C and D). The latter values are expected to represent the docking results for the best ligand pose (supervised docking). For each set we also calculated minimum and maximum values of the FEB and RMSD in order to compare with our control docking simulation. Based on the average and the standard deviations of the FEB values, we do not see much difference between the FFR and the rigid 1ENY models. The only exception is for the PIF ligand. The average best FEB for the initial PIF position was -9.0 ± 2.0, -10.2 ± 1.5, and -10.9 ± 1.4 kcal/mol while for the reference pose, the average FEB was -6.5 ± 2.7, -8.3 ± 2.6 and -8.7 ± 2.3 kcal/mol for InhA_wt, InhA_I16T and InhA_I21V, respectively. The equivalent FEB values for the 1ENY rigid model was -13.4 and -13.5 kcal/mol, respectively. These values represent a difference that varies from 2.5 to 7.0 kcal/mol between the rigid and FFR models of InhA. Also, they are greater than the intrinsic error (2.2 kcal/mol) attributed to the estimation of the FEB by AutoDock3.0.5 [30]. The explicit flexibility of the InhA receptor clearly had an impact in the way PIF interacted with it.

**Table 2:** Summary of docking results for the FFR models of WT, I16V, and I21T InhA enzyme from *M. tuberculosis*

		InhA_WT				InhA_I16T				InhA_I21V			
	
		A	B	C	D	A	B	C	D	A	B	C	D
	Avg	-9.6	5.4	-9.1	4.1	-9.2	4.3	-8.7	3.3	-9.1	5.9	-8.6	3.3
	SD	0.4	2.1	0.5	1.3	0.3	1.6	0.4	0.5	0.3	2.9	0.3	0.8
**ETH**	Min	-11.0	1.9	-10.9	1.4	-10.3	2.2	-10.2	1.7	-10.5	2.4	-9.6	1.7
	Max	-8.4	14.9	-7.5	7.8	-8.2	12.9	-7.4	6.9	-8.4	14.9	-7.2	7.1
	**1ENY**	-9.2	6.0	-8.5	1.8	--	--	--	--	--	--	--	--

	Avg	-12.2	5.6	-10,5	4.3	-11.5	4.6	-10.8	3.3	-11.3	5.2	-10.3	3.2
	SD	0.6	1.8	1.1	1.2	0.5	1.3	0.7	0.4	0.5	1.4	0.5	0.5
**TCL**	Min	-14.3	3.0	-13.5	2.2	-13.0	2.6	-12.7	2.1	-12.9	2.6	-12.0	1.7
	Max	-8.1	14.9	-5.2	13.9	-10.0	10.1	-7.6	6.2	-9.5	9.5	-6.8	6.4
	**1ENY**	-10.7	1.3	-10.6	1.3	--	--	--	--	--	--	--	--

	Avg	-9.0	9.0	-6.5	6.0	-10.2	6.9	-8.3	5.3	-10.9	4.8	-8.7	3.5
**PIF**	SD	2.0	4.0	2.7	3.6	1.5	2.8	2.6	2.4	1.4	2.4	2.3	1.1
	Min	-14.0	3.0	-13.6	2.4	-14.2	3.1	-14.1	2.9	-14.4	2.7	-14.0	2.4
	Max	-1.0	20.0	0.0	16.3	-3.8	18.1	0.0	12.1	-5.0	17.0	-0.1	15.1
	**1ENY**	-13.4	0.9	-13.5	0.3	--	--	--	--	--	--	--	--

The first column indicates the ligands. The second column describes the statistics evaluated for each docking result: the average, standard deviation (SD), the minimum and maximum FEB values. Column 3 (A) contains the values of best FEB in kcal/mol and column 4 (B) its related RMSD in Å. Columns 5 (C) and 6 (D) show the FEB corresponding to the lowest RMSD calculated with respect to the reference ligand position (see Methods section). Columns 3-6 contain the results for InhA_wt. Columns 7-10 and 11-14 are the values of A, B, C and D for InhA_I16T and InhA_I21V mutants, respectively. The last row shows the results for the 1ENY rigid model.

### Analyses of FFR models-ligands interactions

The coordinates of the FFR models-ligands complexes were analysed by LIGPLOT. Through this analysis we were able to identify which residues were making HHB and NNB contacts with the ligands in at least one of the FFRs models’ snapshots (Table 3) which means that, in order to identify those residues we simply considered all snapshots making at least one contact, either HHB or NNB, with each ligand. When the same residue made both intermolecular contacts, the redundancy was eliminated. As Table 3 shows, there is a significant increase in the number of residues that are able to interact with the ligands in the FFR models as compared to the rigid one. Overall, a minimum of nine and a maximum of 80 different residues interacted with any of the three ligands in all FFR models. Conversely, in the rigid 1ENY model these numbers vary from only zero to five. These data further corroborate our hypothesis that considering the explicit flexibility of the receptor during docking simulations allows a ligand to interact, even casually, with other receptor residues than those found in a single, rigid crystal structure. The behaviour of the FFR models simulates the induced fit phenomenon [4].

**Table 3:** Comparison of the number of different amino acid residues making HHB and NNB contacts with the ligands in at least one of the FFR models’ snapshots during the docking simulations

Model	Ligand	Best FEB (HHB)	Best FEB (NNB)	Residues sum (HHB+NNB)	Best Pose (HHB)	Best Pose (NNB)	Residues sum (HHB+NNB)
WT	ETH	52	74	**80**	38	54	**62**
	TCL	25	46	**46**	12	24	**24**
	PIF	23	35	**35**	22	32	**34**

I16T	ETH	34	47	**49**	23	37	**37**
	TCL	19	34	**34**	9	24	**24**
	PIF	23	28	**28**	17	22	**22**

I21V	ETH	31	52	**56**	21	37	**37**
	TCL	18	40	**40**	11	24	**25**
	PIF	22	20	**22**	21	22	**24**

1ENY	ETH	2	4	**5**	1	3	**4**
	TCL	0	1	**1**	0	2	**2**
	PIF	2	2	**2**	0	2	**2**

The total number of receptor residues that made HHB and NNB contacts with the ligands and the union of these residues (HHB+NNB) for the Best FEB (column 5) and for the reference or Best Pose (column 8).

From the data in Table 3 we were able to single out the top 19 FFR amino acid residues that made contact with ETH and PIF ligands, and the top 18 FFR amino acid residues that made contact with TCL, in at least one of their snapshots (Table 4).

**Table 4:** Top 19 and 18 amino acid residues that interacted with the ligands in at least one snapshot for each of the three FFR models during the docking simulations

(a)	InhA_WT - ETH				InhA_I16T - ETH				InhA_I21V - ETH			
Residue	Best FEB		Best Pose		Best FEB		Best Pose		Best FEB		Best Pose	
	
	HHB	NNB	HHB	NNB	HHB	NNB	HHB	NNB	HHB	NNB	HHB	NNB

GLY14	**681**	**363**	64	47	89	80	2	2	**1,652**	**311**	264	18
ILE15	12	164	0	0	81	41	0	0	**487**	39	0	0
SER20	0	**856**	0	100	3	0	0	6	2	99	0	0
ILE21	0	**1,015**	0	**1,974**	0	**971**	0	**1,528**	0	0	0	0
VAL21	0	0	0	0	0	0	0	0	0	**714**	0	**1,022**
PHE41	0	4	0	0	0	65	0	0	0	**426**	0	0
SER94	**1,115**	**955**	**387**	**740**	**1,311**	**516**	156	**762**	**1,712**	**1,683**	**447**	**1,060**
ILE95	**323**	**389**	**392**	229	**713**	**479**	74	**424**	110	**1,566**	230	**617**
GLY96	178	169	2	213	8	**781**	0	124	2	270	0	170
MET147	10	**1,127**	49	**1,805**	3	**755**	9	**943**	2	**849**	3	**1,096**
ASP148	78	**394**	**556**	**587**	**328**	**512**	**842**	**348**	90	104	**444**	**537**
PHE149	5	**1,028**	0	**1,235**	0	**1,529**	0	**1,531**	0	**564**	0	**1,453**
MET161	0	**1,064**	0	**1,664**	1	**1,204**	0	**1,838**	2	**712**	0	**1,675**
LYS165	0	**405**	0	**719**	0	**531**	0	**618**	0	32	0	161
ALA191	4	184	16	**666**	0	177	12	**707**	42	90	55	**483**
GLY192	**779**	68	**903**	155	**959**	140	**1,586**	136	**564**	210	**1,716**	**384**
PRO193	0	**333**	0	305	0	**398**	0	256	0	**408**	0	**720**
ILE194	275	71	0	221	27	**366**	10	**999**	5	12	73	185
THR196	71	216	4	**322**	0	**644**	0	**889**	2	**363**	0	**693**

(b)	InhA_WT - TCL				InhA_I16T - TCL				InhA_I21V - TCL			

Residue	Best FEB		Best Pose		Best FEB		Best Pose		Best FEB		Best Pose	
	
	HHB	NNB	HHB	NNB	HHB	NNB	HHB	NNB	HHB	NNB	HHB	NNB

GLY14	**861**	**934**	**523**	59	101	**533**	18	5	**1,157**	**1,131**	**582**	114
ILE16	0	**494**	0	85	0	0	0	0	0	**571**	0	191
THR16	0	0	0	0	0	**433**	0	38	0	0	0	0
ILE21	0	**998**	0	**2,067**	0	**944**	0	**1,060**	0	0	0	0
VAL21	0	0	0	0	0	0	0	0	0	**638**	0	**1,200**
PHE41	0	47	0	0	0	**519**	0	0	0	**1,383**	0	5
SER94	**1,746**	**1,676**	**1,780**	**1,340**	**1,450**	**605**	**1,213**	67	**638**	**458**	**777**	297
ILE95	241	**562**	**342**	**450**	**645**	**1,134**	259	108	**438**	**1,022**	249	242
GLY96	**370**	**897**	49	**745**	**517**	**1,148**	64	**831**	197	**970**	19	**665**
PHE97	26	71	13	103	17	31	26	**1,430**	2	70	7	125
MET103	2	9	0	0	0	0	0	0	13	33	105	**1,370**
MET147	121	**541**	137	**910**	13	**507**	8	268	13	257	8	**341**
PHE149	10	97	1	287	0	3	0	0	0	2	0	0
MET161	30	**1,262**	13	**2,310**	9	**1,234**	13	**1,872**	3	**322**	13	**1,402**
LYS165	0	176	0	**570**	0	111	0	203	0	0	0	2
GLY192	**1,418**	0	**1,959**	2	**860**	0	**1,308**	0	297	0	**1,302**	1
ILE194	1	0	8	3	2	152	6	**1,830**	13	0	21	17
THR196	16	55	40	269	0	276	6	**438**	2	86	1	**499**

(c)	InhA_WT - PIF				InhA_I16T - PIF				InhA_I21V - PIF			

Residue	Best FEB		Best Pose		Best FEB		Best Pose		Best FEB		Best Pose	
	
	HHB	NNB	HHB	NNB	HHB	NNB	HHB	NNB	HHB	NNB	HHB	NNB

GLY14	83	202	57	103	1	125	2	0	65	**818**	15	**379**
ILE16	0	**765**	0	595	0	0	0	0	0	228	0	147
THR16	0	0	0	0	0	**701**	0	**846**	0	0	0	0
ILE21	0	**456**	0	**1,370**	0	**333**	0	**865**	0	0	0	0
VAL21	0	0	0	0	0	0	0	0	0	**669**	0	**1,547**
ASP42	14	10	1	10	**671**	**553**	53	0	17	13	2	7
PHE93	1	**1,050**	0	0	0	0	3	7	0	0	0	0
SER94	238	**386**	174	**1,047**	170	297	71	**1,043**	47	**1,223**	79	**1,952**
ILE95	2	**773**	9	**1,779**	191	**565**	65	**1,275**	0	**1,942**	1	**2,202**
GLY96	**316**	**1,252**	130	**2,389**	**911**	**1,110**	270	**1,894**	**1,532**	**1,325**	**333**	**2,291**
PHE97	1	**1,050**	7	**1,146**	3	**757**	0	**531**	1	186	0	166
PHE149	0	246	0	**640**	0	121	0	0	0	58	0	78
MET161	0	**494**	1	**1,491**	3	**854**	1	**1,021**	0	**1,436**	5	**1,739**
LYS165	0	160	0	303	0	**347**	0	0	0	12	0	20
GLY192	279	1	**759**	3	189	9	**661**	14	**468**	21	**886**	31
ILE194	11	5	55	7	4	67	9	**335**	17	78	25	**355**
THR196	4	**472**	14	**1,295**	6	**905**	10	**1,491**	38	**2,168**	16	**2,572**
LEU197	3	**430**	0	167	4	285	0	0	5	14	0	7
ALA198	0	**382**	0	**735**	0	105	0	0	1	11	0	21

The first columns show, from the total of amino acid residues found in Table 3, the top 19 (18 for TCL) residues that were able to make contacts, HHB and/or NNB, with the ligands (a) ETH, (b) TCL, and (c) PIF, respectively, in at least 10% of the trajectories. Note that each FFR model is a MD simulation trajectory. Columns 2 to 13 show the number of snapshots for each FFR model involved in such contacts (marked in bold).

Note that we are not trying to count the absolute number of contacts. Instead we are counting how many snapshots of each FFR model provided those contacts. For ETH (Table 4a) we found three polar (SER20, SER94, THR196), one acidic and one basic (ASP148, LYS165), and 14 hydrophobic residues. As for TCL (Table 4b) we found three polar (THR16, SER94, THR196), one basic (LYS165), and 14 hydrophobic residues. For PIF (Table 4c) we found two polar (SER94, THR196), one acidic and one basic (ASP42, LYS165), and 15 apolar residues.

**Figure 3:**
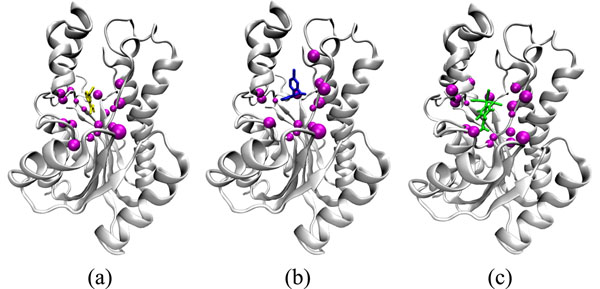
**Top amino acid residues in the receptor-ligand interactions.** Representation of the top 19 and 18 amino acid residues in the receptor structure (PDB ID: 1ENY). The stick representation of ETH (a), TCL (b), and PIF (c) are colored yellow, dark blue, and green, respectively. The residues are represented as magenta beads. The InhA receptor main-chain is represented by ribbons.

As can be seen in column 1 of Table 4, most of the residues are hydrophobic, which is somewhat expected since the InhA active site is predominantly apolar. Figure 3 shows where these residues are located in the receptor structure. These residues are part of both, the NADH and, in particular, the substrate binding pockets.

## Discussion

In order to make molecular docking simulations more realistic, an important issue is to treat both receptor and ligand as flexible structures instead of rigid bodies. However, this has been demonstrated not to be a trivial task [7,14,17,34]. We know that the enzyme InhA from *M. tuberculosis* is a highly flexible receptor [21] and is a very important molecular target for the development of new drugs against tuberculosis [19]. Given that only a few studies [7,12,14,17,34] have addressed the role of the explicit flexibility of receptors in molecular docking simulations and the biomedical importance of the InhA enzyme, here we reported our systematic analysis of the effect of InhA explicit flexibility in docking simulations to three ligands known to be its inhibitors. Our three FFR models of InhA, namely InhA_wt, InhA_I16T, and InhA_I21V were generated from their MD simulations trajectories [21]. As a result we obtained a number of 3-D snapshots of the protein that were slightly different from each other. After that, we then docked each ligand (ETH, TCL and PIF) to each one of FFR models of InhA. We believe that the structural differences from one snapshot to another, creates a space in the receptors binding cavities, which differs from the one we see in the rigid, crystal structure. For instance, in the case of the InhA_wt FFR model, and using the CASTp server [35], the snapshots at 1.0 ns and 1.5 ns have both 1,559 Å^3^ and 1,955 Å^3^, respectively. There is a variation of approximately 400 Å^3^ in the volume of the InhA’s major binding pocket between these two snapshots. This “new space” gives the ligand an opportunity to better explore the binding cavities, increasing its chance to accommodate to it.

Due to the fact that we knew beforehand how ETH and TCL inhibits InhA, blind docking simulations would not be appropriate. ETH binds covalently to carbon 4 of the nicotinamide portion of NADH to form the ETH-NAD adduct [26], whereas the phenolic ring of TCL forms π-stacking interaction with the NADH nicotinamide ring [27,28]. To acknowledge that, in our docking simulations, although the ligands were randomly positioned in the receptor active site, we created what we called the reference ligand. This means that the RMSD calculated during the docking simulations did not use the initial position of the ligand, but rather the position of the reference ligand or Best Pose, closer to the one expected to form the adduct [26]. Our analyses showed that up to 80 different receptors’ residues interact with the ligands as opposed to up to only five residues in the rigid 1ENY model. This constitutes, in our opinion, a strong basis to recommend the use of explicitly flexible models of Mtb’s InhA enzyme in virtual screening efforts to search for novel drug candidates against tuberculosis.

## Conclusions

With our data analyses we were able to find a total of up to 80 receptor amino acid residues interacting with the ligands employed in this study. Performing docking under the same conditions, but in the rigid, crystal structure 1ENY, we were able to find only five for ETH and two for both TCL and PIF. These numbers supports our hypothesis that flexible receptor models can accommodate a more diverse range of ligand conformations. This indicates that they are more prone to select a new ligand capable of binding to InhA than they would do if we used only one receptor conformation. In other words, taking the receptor plasticity into account when performing docking simulation means that amino acid residues, loops and turns, can move slightly in different directions, giving the ligand a better chance to accommodate itself in the receptors’ binding site. Nonetheless, we are aware that our results were based on a short MD simulation; only 3.1 ns long and that nowadays much longer simulations can be generated. Hence, one of our future goals is to consider whether or not longer MD simulations would affect our conclusions, or even how our docking simulations would behave if they were performed on different FFR models generated from MD simulations at physiological temperatures.

## Competing interests

The authors declare that they have no competing interests.

## Authors' contributions

EML, KSM, and ONS conceived and designed the experiments. EML performed the experiments, analyzed the data and wrote the manuscript first draft. MC wrote and executed the automating processing scripts (*Bash*, *Awk* and *Python*). ONS wrote the final version of the manuscript. All authors read and approved the final version of the manuscript.
